# Immune-checkpoint HLA-G gene polymorphisms, 3’-UTR types and their association with hepatocellular carcinoma and treatment response in Indian population

**DOI:** 10.3389/fimmu.2024.1459749

**Published:** 2024-12-05

**Authors:** Neeti Nadda, Renu Yadav, Neelanjana Roy, Nita Singh, Sonu Kumar, Shashi Bala Paul, Shivanand Gamanagatti, Anoop Saraya, Shalimar, Baibaswata Nayak

**Affiliations:** ^1^ Department of Gastroenterology, All India Institute of Medical Sciences, New Delhi, India; ^2^ Department of Radiodiagnosis, All India Institute of Medical Sciences, New Delhi, India

**Keywords:** HLA-G, HCC, genetic association, SNPs (single nucleotide polymorphism), therapeutic response, UTR

## Abstract

**Background:**

Human leukocyte antigen-G (HLA-G) is a cancer-associated immune checkpoint protein implicated in tumor-driven immune escape mechanisms. This study was undertaken to determine genetic variations at the 3’-UTR of the HLA-G gene that may alter its expression, identify risk alleles and genotypes for their association with hepatocellular carcinoma (HCC), and treatment responses in the Indian population.

**Objectives:**

Case-control genetic association study of HLA-G gene UTR polymorphisms with HCC and response to locoregional therapy (LRT).

**Methods:**

HCC cases (n = 100) and healthy controls (n = 110) were recruited for the genetic association study, of which 88 patients received LRT. Single nucleotide polymorphisms (SNPs) at the HLA-G 3’-UTR gene were genotyped by sequencing and PCR-RFLP. The genetic association of 14 SNPs with HCC and LRT responses was determined using population genetic approaches.

**Results:**

Three of the 14 SNPs (rs1707, rs1710, and rs1063320) were found to be genetically associated with HCC risk and treatment responses. These three UTR SNPs are important for miRNA binding. We did not observe significant association of the most studied SNP, rs371194629 (INDEL, +2960), with HCC or treatment response. Serum sHLA-G levels were found to be significantly (p = 0.027) higher in HCC patients as compared to healthy controls. Highly prevalent UTR haplotypes in Indian HCC patients were UTR-4, -1, and -7 whereas in healthy controls it was UTR-3, and 15 as determined by a linkage disequilibrium (LD) plot using 8 SNPs.

**Conclusion:**

HLA-G SNPs are genetically associated with HCC and treatment response. Haplotypes associated with high levels of HLA-G expression are more prevalent in HCC than in healthy controls.

**Core tip:**

Population genetic approaches were used to study HLA-G gene polymorphisms in the Indian population for its genetic association with HCC risk, treatment response and altered gene expression. Out of the 14 SNPs studied for HLA-G UTR, three were linked to HCC and response to locoregional therapy. Linkage disequilibrium and UTR haplotyping analysis show that the UTR-4 haplotype linked to high HLA-G levels, is more common in HCC patients, while the UTR-3 haplotype, linked to low HLA-G levels, is more common in healthy controls. This study is the first to look at the UTR types based on HLA-G gene polymorphisms of Indian HCC patients and their response to therapy.

## Introduction

Human leukocyte antigen-G (HLA-G) is a non-classical human HLA class I molecule, now considered an immune-checkpoint protein due to its role in tumor-driven immune escape mechanisms (1). Tumor cells employ HLA-G overexpression to evade host immune surveillance during carcinogenesis (2). This is mediated by the interaction of HLA-G with inhibitory receptors present on immune cells such as natural killer cells, T cells, B cells, dendritic cells, and neutrophils. This negative signaling counteracts the activated host immune response (3). HLA-G aberrant expressions have been observed in a variety of human cancers including cutaneous melanoma, mesothelioma, glioma, hematopoietic and trophoblastic tumors; lymphoproliferative disorders and carcinoma of lung, ovary, endometrium, bladder, breast, kidney, gastric and colorectal carcinoma. Serum levels of sHLA-G from breast cancer patients were significantly raised in comparison with the healthy individuals (4). The soluble form of HLA-G (sHLA-G) can also be detected in the supernatant of body fluids or malignant effusions from cancer patients. Increased soluble HLA-G (sHLA-G) levels have been reported in patients with melanoma, neuroblastoma, lymphoproliferative disorders, breast, ovarian and colorectal carcinoma when compared to healthy controls or subjects with benign neoplasms (2). The major risk factor for HCC is HBV and HCV infection. Altered expression of soluble and membrane bound isoforms of HLA-G is observed during HBV and HCV infection (4). HLA-G is also thought to be linked with fibrosis progression in HCV infection because there is a positive correlation of HLA-G positive cells with the area of fibrosis on tissue sections (5). Recently, Han et al., 2014 have shown correlation of sHLA-G with infection phases and clinical diseases of HBV infection (6). Both HLA-G mRNA and protein expression have been reported in hepatoma cell lines (7). In HCC patients, high HLA-G expression has been observed in patients with early HCC recurrence and over expression is related to overall survival (8).

The HLA-G locus, as compared to the classical HLA class I gene, has shown low variability within its coding region. These variations in the coding regions are represented as HLA-G alleles, and about 50 coding HLA-G alleles are officially recognized by the IMGT/HLA database 2 that encode only 16 different full-length proteins and two truncated null alleles (9). The non-coding 3′ untranslated region (3’-UTR) and 5′ upstream regulatory region (5′-URR) of the HLA-G locus, on the other hand, are very variable. These polymorphisms are represented as single nucleotide polymorphisms (SNPs) or insertion/deletion (INDELs) polymorphisms, which can influence HLA-G expression by modulating mRNA stability, transcriptional, and posttranscriptional regulation (10). These SNPs are genotyped to get allelic information at individual SNP loci. The SNPs are generally biallelic, which means two possible alleles at an individual SNP locus as they are inherited from parents. There will be three possible genotypes (homozygous dominant, homozygous recessive, and heterozygous) at each SNP locus. Individual SNP alleles should not be confused with the HLA-G allele, which reflects a variant containing nucleotide sequence variations across the entire HLA-G locus or coding region as mentioned in the database. HLA-G gene polymorphisms (both SNPs and INDELs) at 3’-UTR or 5’-URR need to be evaluated for their genetic influence on the expression of HLA-G to make them a genetic biomarker for disease predisposition and progression, or response to therapy.

The SNP rs1063320 (+3142, C/G) and the 14 bp Indel polymorphisms (rs371194629, 14 bp-D/I) reported in HLA-G 3’-UTR region are considered important because it can affect mRNA stability, protein production and implicated in pathological conditions (11). HLA-G1 and HLA-G5 isoform expression is dependent on HLA-G 14bp D/I polymorphisms, with decreased concentrations of HLA-G1 and HLA-G5 in 14 bp insertion samples in comparison with 14 bp deletion samples (12). The +3142 G allele creates a binding site for three microRNAs (miRNAs) (miR-148a, miR-148b, and miR-152) reducing soluble protein production (13). These polymorphisms may further play role in pathological conditions like autoimmune and chronic inflammatory diseases. A study on the Chinese population has reported a lower risk of HCC due to heterozygote (D/I) and the homozygote 14-bp insertion (I/I). The HLA-G 14-bp indel polymorphism may serve as a marker for genetic susceptibility to HCC (14). In this study, genetic associations of HLA-G gene exon 8 polymorphisms with HCC and response to locoregional therapy were studied. The genetic variations in the HLA-G gene UTR of Indian populations, including both HCC and healthy control populations, were studied. The polymorphisms or haplotypes that may influence HLA-G expression were evaluated.

## Materials and methods

### Study design

The study design is a prospective observational case-control study to evaluate the genetic association of HLA-G gene UTR polymorphisms with HCC and response to locoregional therapy.

### Setting

This study was carried out in a tertiary care setting at the Department of Gastroenterology, All India Institute of Medical Sciences, New Delhi, India, from July 2015 to August 2019 after obtaining Institute ethics committee (IEC) approval (IEC/NP-228/05.06.2015/RP-37/2015).

### Participants and clinical investigations

A total of 100 HCC patients attending the liver clinic for treatment were enrolled as cases, whereas a total of 110 healthy volunteers attending the institute’s main blood bank were enrolled as healthy controls for this study. The inclusion criteria were consecutive HCC patients of age > 18 years and willingness to enroll in the study with informed written consent (self or relatives). The exclusion criteria for HCC patients were renal failure, sepsis, pregnancy, and refusal to participate. The details of the HCC population are given in the demographic table (**Table 1**). Diagnosis of HCC in patients was carried out as per European Association for the Study of the Liver (EASL) criteria (15, 16). All HCC patients staging was done as per BCLC staging criteria (17, 18). The recruited patients of BCLC stage A or B have undergone locoregional therapy (LRT) which includes radiofrequency ablation (RFA), percutaneous acetic acid ablation (PAI), trans-arterial chemoembolization (TACE) and trans-arterial radioembolization (TARE). All the variables including demographic profile, clinical, radiological (abdominal ultrasound and MPCT/MRI of the liver) and biochemical parameters of all patients were recorded.

**Table 1:** Demographic, biochemical, and clinical profile of study population.

Parameter	HCC (n=100)	Healthy (n=110)
Age (Years) Mean±SD	56.1 ± 10.7	33.48 ± 11.7
Sex (Male, %)	86 (86%)	95 (86.34)
Etiology (n) HBV HCV Alcohol HBV + HCV NASH HVOTO Cryptogenic	38 (38%) 34 (34%) 5 (5%) 5 (5%) 10 (10%) 1 (1%) 7 (7%)	
Bilirubin (mg%)	1.1 ±0.66	0.58 ± 0.25
Total protein (gm %)	7.3±0.72	7.1± 1.02
Albumin (gm %)	3.8±0.63	4.31± 0.68
AST IU/L	70.4±40.2	25.8 ± 9.36
ALT IU/L	52.9±31.1	33.6 ± 13.5
SAP IU/L	288.1±162.5	164.4 ± 71.3
PT (seconds)	14.1±2.5	
INR	1.2±0.24	
Abdominal pain (n)	23 (23%)	
Tumour Size (cm)	4.44 ± 3.08	
Single Tumour (n) Multiple Tumour (n)	58 (58%) 42 (42%)	
Weight loss (n)	29 (29%)	
GI bleed (n)	6 (6%)	
Ascites (n)	21 (21%)	
Hemoglobin (gm %)	11.85 ± 2.08	
Total Leucocyte counts per mm3	5525 ±2924	
Total platelets count per mm3	132.7±86.9	
Blood Urea (mg %)	27.8± 13.6	
Creatinine (mg %)	0.85±0.28	
Alfa-feto protein (ng/ml, range)	29.15 (1.01-100000)	
BCLC Stage (n) A B C D	44 (44%) 46 (46%) 8 (8%) 2 (2%)	
Performance status (n) PST 0 PST 1 PST 2	86 (86%) 11 (11%) 3 (3%)	
CTP Score (n) 1 2 3	76 (76%) 21 (21%) 3 (3%)	
Loco-regional Therapy (n) PAI RFA TACE TARE	n=88 6 (6.8%) 14 (15.9%) 67 (76.1% 1 (1.2%)	
Tumour Response (n) Complete Response Partial Response Progressive Disease	n=88 44 (50%) 21 (23.9%) 23 (26.1%)	

All values are expressed as n (%) or (mean ± SD) unless otherwise specified.

HBV, Hepatitis B virus; HCV, Hepatitis C virus; NASH, Nonalcoholic steatohepatitis; HVOTO Hepatic venous outflow tract obstruction; AST, Aspartate aminotransferase; ALT, Alanine aminotransferase; SAP, Serum alkaline phosphatase; PT, Prothrombin time; BCLC, Barcelona Clinic Liver Cancer; CTP, Child-Turcotte-Pugh; PST, Performance Status; RFA, Radio frequency ablation; PAI, Percutaneous acetic acid injection; TACE, Trans-arterial chemoembolization; TARE, Trans-arterial Radioembolization.

### Sample size

The sample size was calculated as an independent case-control study based on the expected odds ratios using STATCALC software of Epi Info program (19). The ratio of cases to controls was assumed to one or equal number. The statistical power and confidence level was assumed to 80% and 95%, respectively. The minor allele frequency (MAF) reported at 1000 genome project in dbSNP database for SNP rs371194629 (Ins, 0.3941), and SNP rs1063320 (C, 0.4019) was used as expected proportion exposed (minor allele as risk) in controls. Assuming 40% (MAF=0.40) of controls exposed, total sample size was calculated to n=288 and n=124 at expected OR=2 and 3, respectively. Total 210 subjects including HCC cases (n=100) and healthy control (n=110) were included in this study.

### Collection of blood samples

The blood samples were collected from the enrolled HCC patients and healthy control. Prior to the start of therapy for HCC about 5 ml of the venous blood sample from the peripheral vein in the hand or ante-cubital fossa was collected both in plain and EDTA vials for serum/plasma separation by centrifugation at 1,000 x g and 4°C for 15 minutes. The serum sample were stored at -80^0^ C for serological test.

### Loco-regional therapy and monitoring therapeutic response in HCC patients

After baseline evaluation, patients were evaluated for a treatment plan on the basis of BCLC staging, and appropriate LRT, including RFA, PAI, TACE, or TARE, was given to the patients (20). All patients were followed up for one month following the LRT procedure. The post-therapy response was monitored by radiological imaging techniques like MPCT or MRI of the liver. Response evaluation was done according to the mRECIST criteria, which were categorized into complete response (CR), partial response (PR), progressive disease (PD), and stable disease (SD).

### Detection of HLA-G by enzyme linked immunosorbent assay and western blot

The HLA-G concentrations (ng/ml) in HCC and healthy control sera were determined by commercial ELISA as per the manufacturer’s (Elabscience, USA; cat no. E-EL-H1663) protocol. The detection of HLA-G in serum samples was carried out by western blot using an anti-HLA-G monoclonal (87-G) antibody. Briefly, 20 µl of serum in 1 ml of RIPA lysis buffer was incubated with an anti-HLA-G (87-G) antibody for 1 hour and immunoprecipitated with protein G beads. Immunoprecipitated HLA-G1 proteins were separated on SDS-PAGE and transferred to a PVDF membrane. Western blot was carried out using primary antibodies (anti-HLA-G, 87-G) at a 1:1000 dilution and HRPO-conjugated rabbit anti-mouse secondary antibodies at a 1:1000 dilution. The membranes were washed and developed using an enhanced chemiluminescent detection system (Biorad), and the image was taken with Fluorchem M (Cell Biosciences Inc).

### HLA-G UTR SNPs genotyping

Genomic DNA was isolated from whole blood using the DSP Genomic DNA isolation Kit, Qiagen, in an automated extractor Qiasymphony. The primers were designed for HLA-G UTR amplifications using reference sequences NC_000006 (29826967 to 29831130 nt) and NG_029039. All HLA-G gene 3’-UTR SNPs were genotyped by sanger sequencing of the PCR product and visual chromatogram reading. The 3-UTR region was amplified from genomic DNA using primer pairs T7-8657HLAG-F: taatacgactcactatagggTTGAGGGGAACAGGGGACA and SP6- 9206HLAg-R: atttaggtgacactatagaataGCGCAGCCCCATCTACT, which are flanked by the T7 and SP6 promoter sequences. The PCR amplified product was cycle sequenced using T7 and SP6 primers. The rs371194629 genotypes were further confirmed by gel electrophoresis for the size difference of the PCR-amplified product with 14 bp insertions. The PCR primer pair used for this was 3632F-GTGATGGGCTGTTTAAAGTGTCACC and 3841R-GGAAGGAATGCAGTTCAGCATGA.

### Bioinformatic analysis for prediction of microRNA binding site at HLA-G 3’ UTR

The miRNA target site at the whole 3’-UTR was found using the online tool Targetscan Human Release 8.0 (21), which makes predictions about the biological targets of miRNAs by looking for conserved 6–8 mer sites that match the seed region of each miRNA (22). We also determined the effect of polymorphism on the creation of a new miRNA site using the online miRDB software (23). We obtained the 100 nt sequence flanking the SNP from the dbSNP database and used it as the mRNA target sequence (201 nt) for miRNA prediction. We determined the miRNA-binding seed sequence that flanked the SNP site within the region 80-120 nucleotides.

### Statistical analysis and genetic association

Normally distributed continuous variables were expressed as mean ± standard deviation (SD), and categorical variables were expressed as percentages. The levels of HLA-G were determined in the sera of HCC patients and healthy controls and compared by using a t-test for statistical significance. The genetic association of 3’-UTR SNPs with HCC was determined using population based genetic approaches. The distribution of genotypes in the disease and healthy control groups was derived from Hardy-Weinberg equilibrium (HWE). The genetic association with HCC risk and treatment response was calculated as per the dominant, additive, and recessive inheritance models. Odds ratio, 95% CI interval, and p value were derived by the Chi Square test using a 2x2 contingency table in GraphPad Prism 10.1.2 and SNPstat online software. Major alleles and genotypes were denoted as (1) and (11); minor alleles and genotypes were denoted as (2) and (22) and heterozygotes as (12). Haplotype inference as well as linkage disequilibrium (LD), LOD, and r^2^ were analyzed with HAPLOVIEW (version 4.1) (Dr. Mark Daly’s lab, MIT/Harvard Broad Institute).

## Results

### Demographic, biochemical, and clinical profile of the study population

The demographic details were mentioned in **Table 1**. A total of 100 HCC patients with a mean age of 56.1 years were enrolled, and most of the patients were male (86%). Majorities of HCC patients are at early HCC 90% (BCLC stage A, 44% and stage B, 46%) than advanced HCC, 10%. The underlying etiologies of HCC were viral, 72% (HBV, 38%, and HCV, 34%), as compared to non-viral etiologies, 28% (NASH, 10%; cryptogenic, 7%; alcohol, 5%; combined HBV and HCV etiology, 5% and HVOTO, 1%). The average tumor size in HCC patients was 4.44 ± 3.08 cm, and 58% of patients had a single lesion. Locoregional therapy was received by 88 HCC patients, and these included various types of LRTs such as RFA (15.9%), PAI (6.8%), TACE (76.1%), and TARE (1.2%). One-month post-treatment response as per mRECIST criteria was CR (50%), PR (23.9%), and PD (26.1%).

### Soluble HLA-G levels in the sera of HCC patients and healthy control

**Figure 1:**
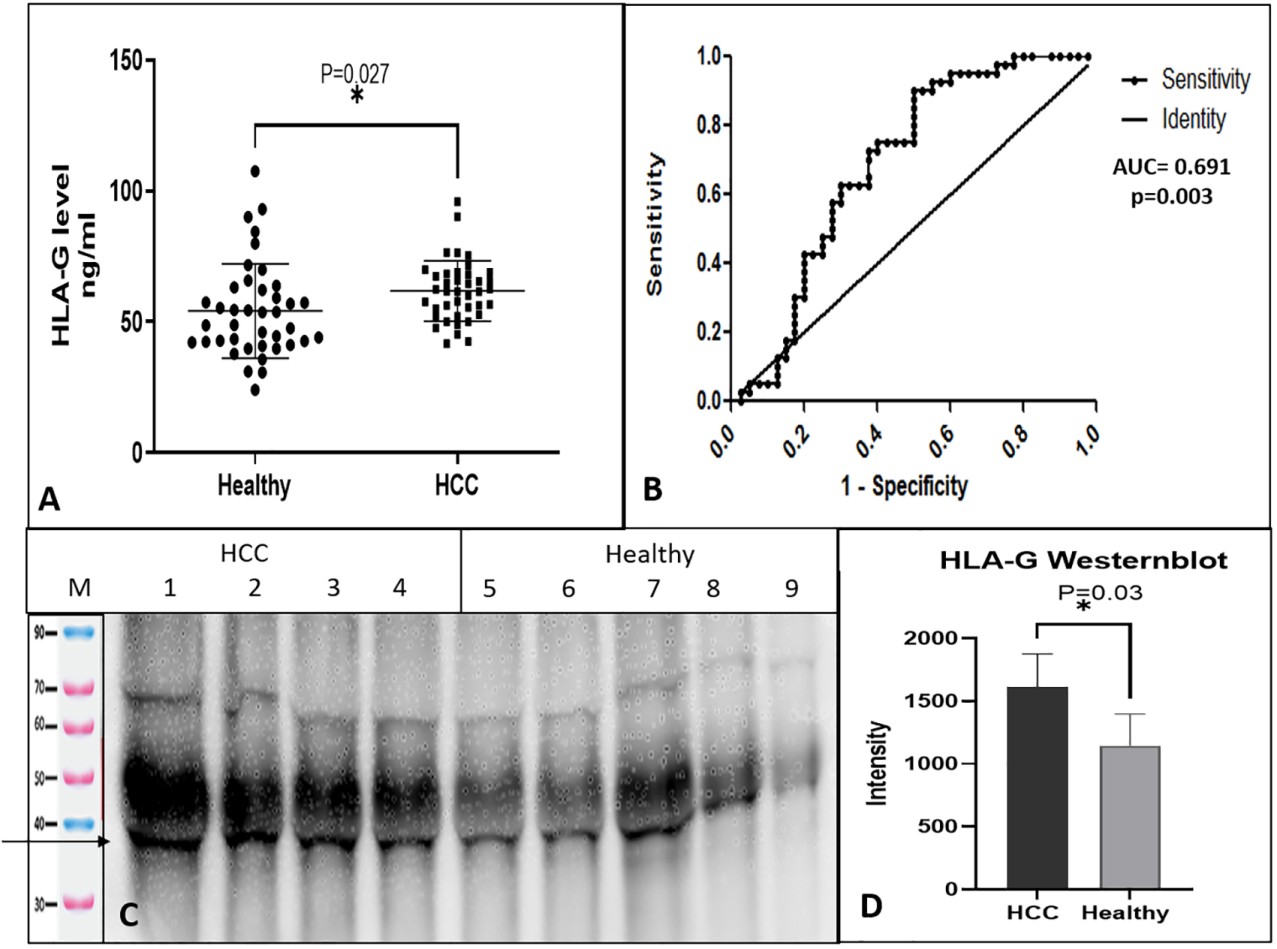
Soluble HLA-G expression in serum and receiver operating characteristic (ROC) curve differentiating HCC patients from healthy control. **(A)** Soluble HLA-G levels in serum of healthy controls and HCC patients, **(B)** ROC curve differentiating HCC patients from healthy control. **(C)** HLA-G expression by western blot of HCC and healthy serum samples. **(D)** Densitometry analysis of westernblot. * significant (P< 0.05).

Levels of soluble HLA-G protein were determined in the sera of HCC patients and healthy controls using the sandwich ELISA test. Total 80 subjects (HCC, n = 40 and healthy controls, n = 40) were tested for sHLA-G. The serum levels of HLA-G were found to be significantly higher (p = 0.027) in HCC patients (61.8 ± 11.5 ng/ml) as compared to healthy (54.2 ± 17.9 ng/ml) controls. **Figure 1A** shows the levels of HLA-G in the serum of HCC as compared to healthy subjects. To differentiate HCC from healthy, an ROC curve was plotted, and the area under the curve was AUC=0.691(95% CI, 0.571-0.811) (**Figure 1B**) with p-value of 0.003. The cut-off value was found to be >55.42 ng/ml at 0.72 (95% CI, 0.561-0.854) sensitivity and 0.625 (0.458-0.772) specificity. This result indicates that HLA-G concentration in serum can differentiate HCC from healthy.

The levels of sHLA-G protein were checked by western blot in the serum of HCC and healthy individuals. The sHLA-G fraction was immunoprecipitated using an anti-HLA-G 87-G antibody and protein-G, which detected a clear band of size 35 KDa (**Figure 1C**). Densitometry analysis indicated higher levels of sHLA-G in HCC patients than healthy subjects (**Figure 1D**).

### Genotyping of HLA-G gene exon 8 single nucleotide polymorphisms

**Figure 2:**
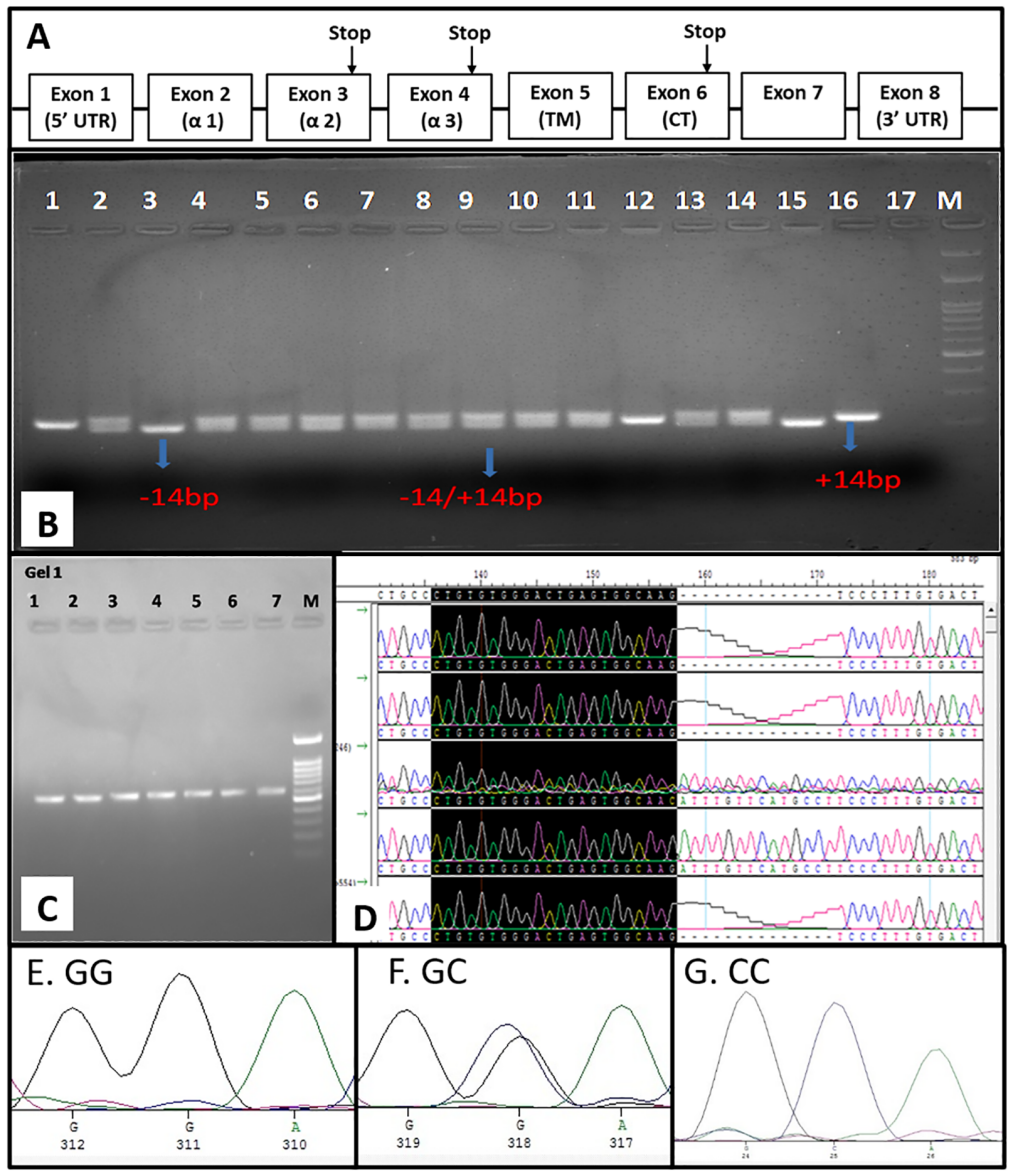
Genomic organization of HLA-G and exon 8 SNP genotyping. **(A)** Genomic organization of HLA-G gene depicting eight exon **(B)** SNP genotyping of INDEL polymorphism by PCR-RFLP **(C)** PCR amplification of Exon 8 for sequencing **(D)** SNP genotyping by visual chromatogram reading INDEL polymorphism showing DEL, INS and DEL/INS genotype and the chromatogram represent homozygous GG **(E)**, heterozygous GC **(F)** and homozygous CC **(G)** for +3142 SNP rs1063320 C>G.

The HLA-G gene consists of 7 introns and 8 exons, as mentioned in **Figure 2A**. The exon 8 region spans the 3’-UTR of HLA-G mRNA. In the HLA-G 3′ UTR locus, +2960 SNP (rs371194629), a 14-base-pair insertion/deletion (14-bp INS/DEL), and +3142 SNP (rs1063320 C/G) were the most studied polymorphisms. All 14 SNPs in the 3’-UTR or exon-8 region were genotyped by sequencing and chromatograph reading. The SNP ID corresponding region were mentioned in **Table 2**. Additionally, 14-bp INDEL polymorphism was genotyped by size differences of the amplified PCR product when run through 3% agarose gel electrophoresis (**Figure 2B**). The amplified product of 210-bp is for homozygous (DD, -14bp) deletion, and the product size is 224bp for homozygous insertion (Ins/Ins, II +14bp). Two different PCR products of sizes: (210 and 224 bp) represented heterozygous genotypes (Del/Ins, -14/+14 bp). For SNP genotyping by the sequencing method, the 3’-UTR flanking the 8790 nt - 9144 nt region (549 bp) was PCR amplified (**Figure 2C**). These PCR products were sequenced using T7 forward and SP6 reverse primers as amplicons flanked with T7 and SP6 promoter sequences. The chromatogram showing +2960 SNP (rs371194629) in 5 patients is depicted in **Figure 2D**. Samples 1, 2, and 5 show deletion, whereas samples 3 and 4 show Del/Ins and Ins, respectively. The chromatogram of +3142 SNP (rs1063320, C>G) for homozygous GG, heterozygous GC and homozygous CC genotype are depicted in the **Figures 2E–G**, respectively. The genomic sequences were submitted to GenBank with accession number spanning from PP278638 to PP278737 for HCC patients and the accession number spanning from PP278738 to PP278847 for healthy controls.

**Table 2:** HLA-G SNP genotype and allele frequency distribution and Hardy-Weinberg equilibrium testing.

SNPs ID, Chr 6, UTR position	Genotype, Minor Allele	HCC N=100	HWE P value	Healthy N=110	HWE P value	MAF Range (1000 genome)
**SNP1: rs371194629** DEL (D)> INS (I) Chr 6:29830804 UTR: 2960	DD DI II I	31 (0.31) 50 (0.50) 19 (0.19) 0.44	0.8838	25 (0.227) 62 (0.564) 23 (0.209) 0.49	0.1806	ATTTGTTCATGCCT (0.18-0.45) INS=0.3936
**SNP2 : rs567747015** C>T Chr 6:29830831 UTR : 3001	CC CT TT T	74 (0.74) 26 (0.26) 0 0.13	0.1351	83 (0.754) 27 (0.246) 0 0.12	0.1423	(0.003-0.006) T=0.0068
**SNP3 : rs1707** (C>T) Chr 6:29830840 UTR: 3003	TT TC CC C	50 (0.50) 24 (0.24) 26 (0.26) 0.38	**0.000**	73 (0.664) 21 (0.182) 16 (0.154) 0.24	**0.000**	(0.004-0.219) C=0.0943
**SNP4:rs1710** (G>C) Chr 6:29830840 UTR:3010	CC GC GG G	36 (0.36) 15 (0.15) 49 (0.49) 0.57	**0.000**	60 (0.555) 17 (0.145) 33 (0.30) 0.38	**0.000**	(0.236-0.486) G=0.400
**SNP5: rs17179101** (C>A) Chr 6:29830857 UTR: 3027	CC CA AA A	85 (0.85) 3 (0.03) 12 (0.12) 0.14	**0.000**	95 (0.864) 0 15 (0.136) 0.14	**0.000**	(0.031-0.085) A=0.085
**SNP6: rs146339774** (G>C) Chr 6:29830862 UTR: 3032	GG CG CC C	98 (0.98) 1 (0.01) 1 (0.01) 0.02	**0.000**	108 (0.982) 2 (0.018) 0 (0.0) 0.01	0.923	(0.001-0.004) C=0.0014
**SNP7 : rs17179108** (C>T) Chr 6:29830865 UTR: 3035	CC CT TT T	83 (0.83) 3 (0.03) 14 (0.14) 0.16	**0.000**	90 (0.791) 4 (0.045) 16 (0.164) 0.16	**0.000**	(0.047-0.1759) T=0.145
**SNP8 : rs569057854** C>T Chr 6:29830882 UTR: 3052	CC CT TT T	99(0.99) 1(0.01) 0(0.0) 0.01	0.9599	110 (1.0) 0 (0.0) 0(0.0) 0	Monomorphic	(0.0009-0.0012) T=0.0010
**SNP9 : rs180827037** (G>T) Chr 6:29830922 UTR: 3092	GG GT TT T	100 (1.0) 0 (0.0) 0 (0.0) 0	Monomorphic	110 (1.0) 0 (0.0) 0 (0.0) 0	Monomorphic	(0.0-0.009) T=0.00031
**SNP10: rs760052251** G>A Chr 6:29830950 UTR:3120	GG GA AA A	100 (1.0) 0 (0.0) 0 (0.0) 0	Monomorphic	110 (1.0) 0 (0.0) 0 (0.0) 0	Monomorphic	A=0.0001-0.0003 NA
**SNP11 : rs138249160** T>C Chr 6:29830951 UTR: 3125	TT TC CC C	91 (0.91) 9 (0.09) 0 (0.0) 0.05	**0.000**	96 (0.846) 14 (0.145) 0 (0.0) 0.06	0.476	(0.00006-0.013) C=0.0012
**SNP12: rs1063320** C>G Chr 6:29830972 UTR: 3142	CC GC GG G	54 (0.54) 18 (0.18) 28 (0.28) 0.37	**0.000**	41 (0.454) 19 (0.164) 50 (0.382) 0.54	**0.000**	(0.236-0.495) C=0.4002
**SNP13: rs9380142** A>G Chr 6:29831017 UTR: 3187	AA AG GG G	73(0.76) 9 (0.09) 13(0.13) 0.184	**0.000**	78 ( 0.76) 10 (0.09) 14 (0.137) 0.186	**0.000**	(0.23-0.45) G=0.2535
**SNP14:rs1610696** C>G Chr 6:29830972 UTR: 3196	CC CG GG G	91(0.95) 1 (0.01) 3(0.03) 0.036	**0.000**	97 (0.95) 0 5(0.05) 0.049	**0.000**	(0.08 – 0.5) G=0.250

The bold values in HWE p-value column indicate significant (p<0.05) value.

### HLA-G 3’-UTR/exon-8 SNP genotype and minor allele frequency distribution

In our current study, we have studied the allele properties of 14 HLA UTR SNPs in the Indian population among HCC patients as cases and healthy subjects as controls. These SNPs were SNP1: +2960, rs371194629 DEL/INS, SNP2: +3001, rs567747015 (C>T), SNP3: +3003, rs1707 (C>T), SNP4: +3010, rs1710 (G>C), SNP5: +3027, rs17179101 (C>A), SNP6: +3032, rs146339774(G>C), SNP7: +3035, rs17179108(C>T), SNP8: +3052, rs569057854 (C>T), SNP9: +3092, rs180827037 (G>T), SNP10: +3120, rs760052251 (G>A), SNP11: +3125, rs138249160 (T>C), SNP12: +3142, rs1063320 (C>G), SNP13: +3187, rs9380142 (A>G) and SNP14: +3196, rs1610696 (C>G). For this, we have evaluated both genotype and allele frequencies in HCC and healthy Indian control population and evaluated whether they are in Hardy-Weinberg equilibrium or not (**Table 2**). The distributions of SNP1 rs371194629 Del/INS genotypes DD, DI, and II were 31%, 50%, and 19% for HCC cases (n =100), and 22.7%, 56.4%, and 20.9% for healthy controls (n=110), respectively. The test of deviation from Hardy-Weinberg equilibrium was not found to be significant for four SNPs: +2960 SNP1 (rs371194629, Del >Ins), +3001 SNP2 (rs567747015, C>T), +3032 SNP6 (rs146339774, G>C), and +3125 SNP11 (rs138249160, T>C) in the healthy control population. Three SNPs, including +3032 SNP8 (rs569057854, C>T), +3092 SNP9 (rs180827037, G>T), and +3120 SNP10 (rs760052251, G>A), were found to be monomorphic in the control population, whereas only SNPs 9 and 10 were monomorphic in the HCC population.

### Tests for association of HLA SNP genotype and allele with HCC

The genetic variations at each SNP include major (1) and minor (2) alleles that result in three genotypes: major allele homozygote (11), heterozygote (12), and a minor allele homozygote (22) genotype. The impact of each SNP variant (both genotypes and alleles) or its genetic association with disease (HCC) risk in the Indian population was studied in this case-control study. The strength of association for each genotype and allele was derived from population frequency data using a two-by-two contingency table for the estimation of an odds ratio (OR) and 95% confidence intervals (CI). The OR was estimated by transforming three genotypes into two variables using different inheritance models that include the co-dominant model: heterogeneous (11 vs. 12) and homogeneous (11 vs. 22); additive models: dominant (11 vs. 12 + 22), recessive (22 vs. 12 + 11), and allelic (1 vs. 2) models (**Table 3**). The statistical significance of OR was obtained at a 95% confidence interval, and the p-value was analyzed by Fisher’s exact test, assuming minor allele (2) and genotype (22) as risk and dominant allele (1) and genotype (11) as references (**Table 3**). Statistically significant (P<0.05) association of SNP is considered risk of disease or HCC when OR>1 and 95% CI lower limit is ≥ 1, whereas SNP is considered protective when OR<1 and 95% CI upper limit <1.

**Table 3:** Genetic association of HLA-G 3’-UTR polymorphisms with HCC patients as compare to healthy control.

Genotype, Allele	OR, 95 % CI, Risk allele 2	P value	Genotype, Allele	OR, 95 % CI, Risk allele 2	P value
**SNP1: rs371194629,** DEL (D)> INS (I), +2960			SNP7 :rs17179108,(C>T), +3035		
DD (11) DI (12) II (22) DI+II (12+22) DI+DD(12+11) D allele (1) I allele (2)	Ref 0.65 [0.34-1.24] 0.66 [0.29-1.48] 0.65 [0.35-1.21] 1.12 [0.57-2.22] Ref 0.81 [0.55-1.19]	- 0.19 (Ref: DD) 0.32 (Ref: DD) 0.17 (Ref: DD 0.72 (Ref:II) - 0.29 (Ref : D)	CC CT TT CT+TT (12+22) CT+CC (12+11) C allele T allele	Ref 0.81 [0.20-3.10] 0.95[0.45-2.10] 0.92[0.46-1.91] 1.04[0.47-2.18] Ref 0.93 [0.56-1.60]	- 0.99 (Ref :CC) 0.99 (Ref :CC) 0.86(Ref :CC) 0.99 (Ref:TT) - 0.89 (Ref :C)
SNP2: rs567747015, C>T, +3001			**SNP11 :rs138249160**T>C, +3125		
CC (11) CT (12) TT (22) CT+TT (12+22) CT+CC (12+11) C allele (1) T allele(2)	Ref 1.08 [0.58-1.99] - - - Ref 1.06 [0.60-1.88]	- 0.87 (Ref : CC) - - - - 0.88 (Ref :C)	TT (11) TC(12) CC (22) TC+CC (12+22) TC+TT (12+11) T allele (1) C allele (2)	Ref 0.68[0.28-1.65] - 0.68[0.28-1.65] - - 0.69 [0.27-1.60]	- 0.507 (Ref :TT) - 0.507 (Ref :TT) - - 0.52 (Ref :T)
**SNP3 : rs1707,** (C>T), + 3003			**SNP 12: rs1063320, C>G, +**3142		
TT (11) CT (12) CC (22) CT+CC (12+22) CT+TT (12+11) T allele (1) C allele (2)	Ref 1.66 [0.84-3.34] **2.37 [1.15-4.87]** **1.97 [1.13-3.36]** 0.83 [0.37-1.71] Ref **1.93 [1.25-2.95]**	- 0.16 (Ref: TT) **0.02 (Ref:TT)** **0.01 (Ref:TT)** 0.70 (Ref: CC) - **0.002 (Ref: T)**	GG (11) GC (12) CC (22) GC+CC (12+22) GC+GG (12+11) G (1) C (2)	Ref 1.69[0.73-3.59] **2.35[1.26-4.33]** **2.14[1.19-3.84]** **0.50 [0.28-0.87]** Ref **2.00[1.35-2.94]**	0.224(Ref :GG) **0.009**(Ref :GG) **0.010** (Ref :GG) **0.018** (Ref :CC) - **0.000** (Ref :G)
**SNP 4: rs1710,** (G>C), +3010			SNP13: rs9380142, A>G, +3187		
CC (11) GC (12) GG (22) GC+GG (12+22) GC+CC (12+11) C allele (1) G allele (2)	Ref 1.47[0.66-3.17] **2.47[1.35-4.45]** **2.13[1.23-3.63]** **0.44 [0.25-0.78]** Ref **2.14 [1.44-3.14]**	- 0.40 (Ref: CC) **0.004 (Ref:CC)** **0.008(Ref:CC)** **0.04 (Ref: GG)** - **0.001 (Ref:C)**	AA (11) AG (12) GG (22) AG+AA (12+22) AG+GG (12+11) A allele (1) G allele (2)	Ref 0.96[0.39-2.60] 0.99[0.43-2.22] 0.97[0.51-1.86] 0.98 [0.38-2.58] Ref 0.98 [0.58-1.63]	- 0.99 (Ref :AA) 0.99 (Ref :AA) 0.99(Ref :AA) 0.99 (Ref :GG) 0.99(Ref :A)
SNP5: rs17179101(C>A), +3027			SNP 14:rs1610696, C>G, +3196		
CC (11) CA (12) AA (22) CA+AA (12+22) CA+CC(12+11) C allele (1) A allele (2)	Ref - 0.89 [0.38-1.94] 1.11 [0.51-2.40] 1.25 [0.46-3.44] Ref 0.98[0.57-1.75]	- - 0.83 (Ref: CC) 0.84 (Ref: CC) 0.79 (Ref: AA) - 0.99 (Ref:C)	CC (11) GC (12) GG (22) GC+GG (12+22) GC+CC (12+11) C allele (1) G allele (2)	Ref - 0.63[0.16-2.51] 0.85[0.25-2.95] 1.58 [0.40-6.09] - 0.74 [0.28-2.05]	0.72 (Ref :CC) 0.99 (Ref :CC) 0.723 (Ref :GG) 0.62 (Ref :C)
SNP6: rs146339774 (G>C), +3032					
GG (11) GC (12) CC (22) GC+CC (12+22) GC+GG (12+11) G allele (1) C allele (2)	Ref 0.55 [0.03-4.80] - 1.10[0.17-7.13] - Ref 1.66 [0.33-9.42]	- 0.99 (Ref: GG) - 0.99 (Ref :GG) - - 0.67(Ref :G)			

The bold values indicate significant (p<0.05) association or Odds ratio.

There was no significant association of +2960 SNP1 (rs371194629) Ins-allele and Ins/Ins (I/I)-genotype with HCC risk. Significant associations with HCC risk were observed for +3003 SNP3 (rs1707), +3010 SNP4 (rs1710), and +3142 SNP12 (rs1063320). Assuming dominant TT as the reference genotype, OR was calculated for CT, CC, and CT+CC genotypes. The ORs for +3003 SNP3 genotypes were found to be > 1 (1.66 = OR_CT_, 2.37 = OR_CC_ and 1.97 = OR_CT+CC_), as mentioned in **Table 3**. The lower and upper limits of the 95% confidence interval for the OR were calculated. The lower limit of the 95% CI is 1.25 (OR_C_), 0.84 (OR_CT_), 1.15 (OR_CC_), and 1.13 (OR_CT+CC_). The p values for all except OR_CT_ are significant < 0.05. Assuming minor allele C as an HCC risk and dominant T allele as a protective allele or reference for +3003 SNP3, the OR of the C allele was calculated (OR_C_ = 1.93) and found significant (**Table 3**).

Assuming the major C allele as a reference for +3010 SNP4 (rs1710), the OR was calculated for allele G (OR_G_ = 2.14), which was found to be significant for HCC risk. Also, assuming dominant CC as the reference genotype, OR was calculated for GG and GC+GG genotypes and found significant. OR_GG_ and OR_GC+GG_ were 2.47 and 2.13 with p value of 0.004 and 0.008 respectively.

For +3142 SNP12 (rs1063320), the minor allele is C and the major allele is G. Assuming allele G as a reference, the OR of risk allele C was calculated (OR_C_ = 2.0) and found to be significant. Assuming dominant GG allele as a reference for SNP12, OR_CC_ and OR_GC+GG_ was 2.35 and 2.14 with significant p value <0.05.

### Association of HLA-G UTR polymorphisms with therapeutic response to loco-regional therapy

Out of 100 recruited HCC patients, 88 have undergone locoregional therapies, and their one-month post-therapy response was evaluated by radiological imaging techniques as per mRECIST criteria. The association of HLA-G polymorphisms with therapeutic responses was evaluated using the chi-square test. Response to therapy in HCC patients were categorized as CR, PR, and PD. Two groups [Group 1: CR vs. no-CR (PR + PD) and Group 2: CR+PR (responder) vs. non-responder (PD)] were compared using the chi-square test for genetic association (**Table 4**). The genetic associations of SNPs with response to locoregional therapy in HCC patients were observed for 3 out of 14 SNPs, which include +3003 SNP3 (rs1707), +3010 SNP4 (rs1710), and +3142 SNP12 (rs1063320). Assuming the GG-genotype as a reference, the GC genotype showed a significant (p = 0.04) association for no-CR (PR+PD) with an OR value of 4.26 (1.19-15.26) for +3142 SNP12 (rs1063320). Two SNPs were linked to treatment response in the second group, which was either a responder (response, CR+PR) or a non-responder (PD). The OR_CT_ and OR_CT+CC_ for +3003 SNP3 (rs1707) were 3.86 (95% CI 1.19–12.44) and 3.43 (1.24–9.48), assuming TT as the reference and CC as the risk genotype with significant P value of 0.03 and 0.017. For +3010 SNP4 (rs1710), considering CC as a reference, OR_GC_ was 5.14 (1.33–19.76) with a significant p-value (p = 0.01).

**Table 4:** Association of HLA-G 3’-UTR polymorphisms with treatment response to locoregional therapy in HCC patients.

Response	Complete Response vs No-CR CR vs PR+PD, Risk genotype 22			Responder vs Non-Responder CR+PR vs PD, Risk genotype 22		
Genotype	Yes/No	OR, 95 % CI	P value	Yes/No	OR, 95 % CI	P value
SNP1: rs371194629						
DD (11) DI (12) II (22) DI+II (12+22)	12/16 22/21 10/7 32/28	Reference 0.72(0.27-1.86) 0.53(0.15-1.78) 0.66(0.27-1.62)	- 0.627 0.365 0.492	20/8 30/13 15/2 45/15	Reference 1.08(0.38-3.09) 0.33(0.06-1.80) 0.83(0.30-2.28)	**-** 1.00 0.275 0.796
SNP2: rs567747015						
CC (11) CT (12) TT (22) CT+TT (12+22)	33/29 11/15 - 11/15	Reference 1.55(0.61-3.91) - 1.55(0.61-3.91)	- 0.483 - 0.483	48/14 17/9 - 17/9	Reference 1.8(0.66-4.95) - 1.8(0.66-4.95)	- 0.290 - 0.290
SNP3: rs1707						
TT (11) CT (12) CC (22) CT+CC (12+22)	24/22 9/13 11/9 20/22	Reference 1.57(0.56-4.41) 0.89(0.31-2.56) 1.20(0.51-2.77)	- 0.443 1.00 0.831	39/7 13/9 13/7 26/16	Reference **3.86(1.19-12.44)** 3.00(0.88-10.18) **3.43(1.24-9.48)**	- **0.03** 0.101 **0.017**
SNP4: rs1710						
CC(11) GC(12) GG(22) GC+GG (12+22)	16/17 4/11 24/16 28/27	Reference 2.58(0.68-9.81) 0.63 (0.24-1.59) 0.90(0.38-2.15)	0.212 0.353 1.0	27/6 7/8 31/9 38/17	Reference **5.14(1.33-19.76)** 1.30(0.41-4.14) 2.01(0.70-5.77)	**0.019** 0.774 0.219
SNP5: rs17179101						
CC (11) CA (12) AA (22) CA+AA (12+22)	36/39 1/2 7/3 8/5	Reference 1.85(0.16-21.2) 0.39(0.09-1.64) 0.57(0.17-1.92)	- 1.00 0.313 0.0549	52/23 3/0 10/0 13/0	Reference - - -	–
SNP7: rs17179108						
CC (11) CT (12) TT (22) CT+TT (12+22)	35/38 3/0 6/6 9/6	Reference - 0.92 (0.27-3.12) 0.61 (0.19-1.90)	- - 1.0 0.57	51/22 3/0 11/1 14/1	Reference - 0.21(0.02-1.73) 0.16(0.02-1.33)	- - 0.166 0.103
SNP11:rs138249160						
TT (11) TC (12) CC (22) TC+CC (12+22)	44/44 5/3 - 5/3	Reference 0.6(0.13-2.66) - 0.6(0.13-2.66)	- 0.715 - 0.715	65/23 6/2 - 6/2	Reference 0.9(0.17-5.04) - 0.9(0.17-5.04)	1.00 - 1.00
SNP12: rs1063320						
GG (11) GC (12) CC (22) GC+CC (12+22)	27/19 4/12 13/13 17/25	Reference **4.26(1.19-15.26)** 1.42(0.54-3.73) 2.09(0.89-4.89)	**0.04** 0.62 0.134	35/11 8/8 22/4 30/12	Reference 3.18(0.97-10.47) 0.58(0.16-2.04) 1.27(0.49-3.30)	0.067 0.401 0.694
SNP13: rs9380142						
GG (11) GA (12) AA (22) GA+AA (12+22)	6/6 4/4 31/32 35/36	Reference 1.00(0.16-5.98) 1.03(0.30-3.54) 1.02(0.30-3.49)	1.0 1.0 1.0	10/2 6/2 45/18 51/20	Reference 1.67(0.18-15.14) 2.00(0.39-10.05) 1.96(0.39-9.75)	1.0 0.49 0.50
SNP14: rs1610696						
GG (11) GC (12) CC (22) GC+CC (12+22)	2/1 0/1 39/40 39/41	Reference - 2.05(0.17-23.56) 2.10(0.18-24.14)	- - 1.0 0.615	2/1 0/1 59/20 59/21	Reference - 0.67(0.05-7.88) 0.71(0.06-8.26)	- - 1.0 1.0

The bold values indicate significant (p<0.05) association or Odds ratio.

### Prediction of miRNAs binding to mRNA having changes due to polymorphisms at SNPs rs1707, rs1710, and rs1063320 loci

Target Scan predicted 181 miRNAs binding to the human HLA-G UTR (HLA-G, ensemble ID: ENST00000376828.2 and 3’ UTR length: 386 nt). Target prediction was carried out by miRDB software with an HLA-G mRNA target sequence containing rs1707, rs1710, and rs1063320 SNP alleles. Three miRNAs (miR-654-5p, miR-541-3p, and miR-4492) are predicted to bind mRNA UTR sequences flanking SNP rs1707 containing either the C or T allele. Five miRNAs (miR-654-5p, miR-541-3p, miR-3158-5p, miR-4492, and miR-4498) seed sequences were predicted to bind sequences flanking the rs1710 SNP site. The binding site of miR-3158-5p was predicted alone for the rs1710 C-allele, whereas the rest of the miRNA can bind when SNP flanking either the G or C allele. The miR-4800-5p, miR-7705, miR-3619-3p, and miR-6854-5p can bind to SNP rs1063320 G or C allele, and miR-767-5p is restricted for the rs1063320-C allele.

### Linkage disequilibrium profile of exon 8 or 3’-UTR region SNPs of HLA-G

**Figure 3:**
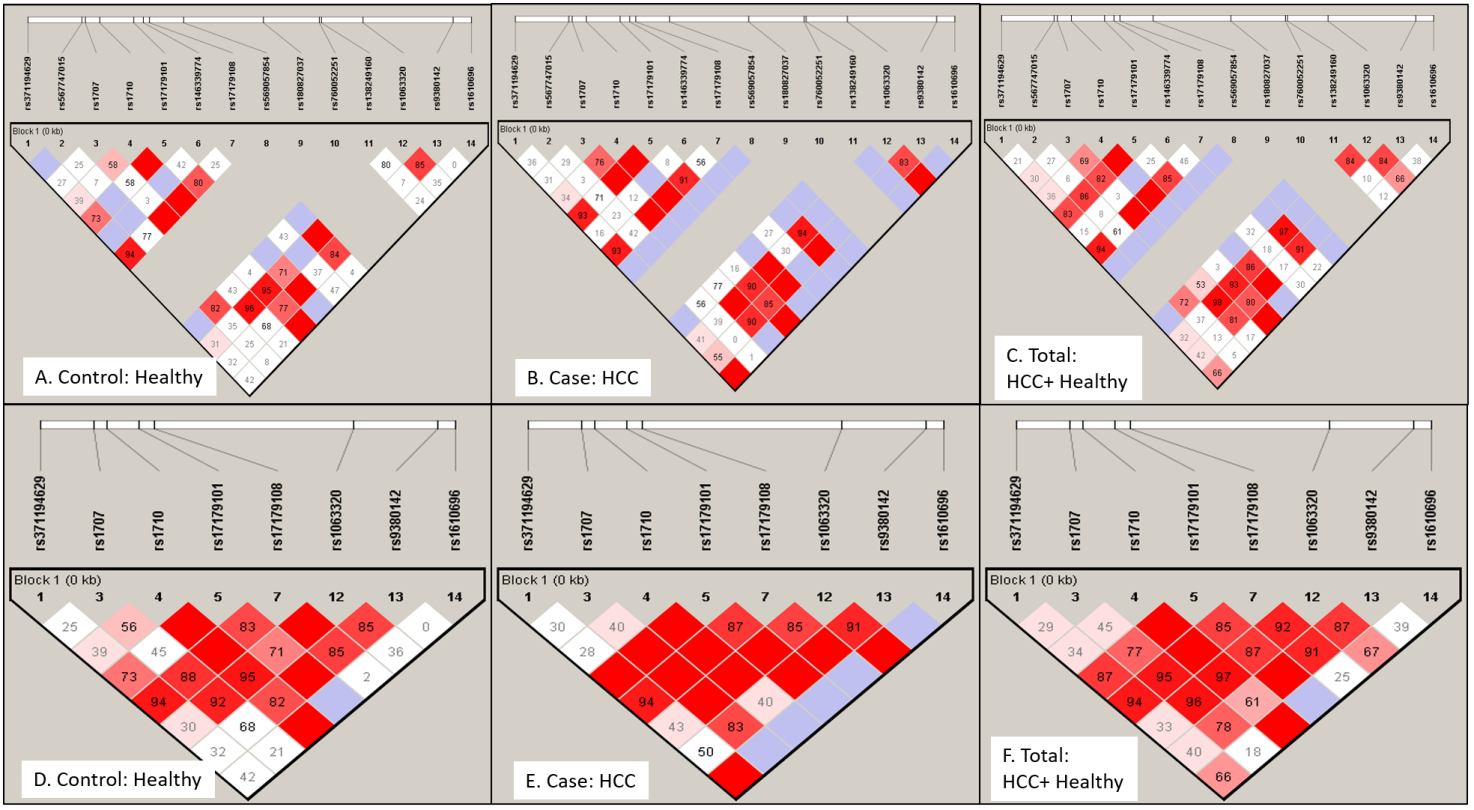
Linkage disequilibrium (LD) plot for HLA-G gene exon 8 or 3’-UTR region SNPs: LD profile using 14 SNPs **(A–C)** and 8 SNPs **(D–F)** were plotted for Healthy control **(A)** and **(D)**, HCC patients **(B)** and **(E)** and total Indian population **(C)** and **(F)**. LD plot indicates non-random association of alleles at different SNP loci. Strong linkage or association is depicted as bright red (D'=1 or 100%, LOD ≥ 2), shades of pink or red (D'<1, LOD ≥ 2), whereas inconclusive LD and no LD are depicted as blue (D'=1, LOD <2) and white (D'<1, LOD <2). Number in block indicates D' value in percentage.

The non-random association of alleles at 14 different SNP loci was anlayzed for linkage disequilibrium (LD) using Haploview software. A pairwise LD value or coefficient of linkage disequilibrium (D’) was used to generate color blocks in the LD plot (**Figure 3**). Strong LD is depicted as bright red (D’=1, LOD ≥ 2), shades of pink or red (D’<1, LOD ≥ 2), whereas inconclusive LD and no LD are depicted as blue (D’=1, LOD <2) and white (D’<1, LOD <2). A high LOD (logarithm of odds) score indicates strong linkage, whereas a high r^2^-value (r^2^ > 0.6) indicates strong correlation between two SNPs. Percentage D’ value is depicted in the colour block of LD plots of healthy (3A, D), HCC (3B, E), and combined population (3C, F) with its LOD score and r^2^ value (3D) for both 14 SNPs (**Figures 3A–C**) and 8 SNPs (**Figures 3D–F**) panel.

In the case of the healthy group (**Table 5**A), LD was observed for +2960 SNP1 (rs371194629) with SNP4(rs1710), SNP5 (rs17179101), SNP7 (rs17179108) and SNP12 at LOD, 3.81,4.67,9.68 and 3.28 respectively. +3001 SNP2 (rs567747015) with SNP11 (rs138249160) at LOD, 6.2; +3003 SNP3 (rs1707) with SNP4, SNP7 (rs17179108) and SNP12 (rs1063320) at LOD, 7.61, 3.87 and 17.18; +3010 SNP4 (rs1710) with SNP5, SNP7, SNP12, SNP13, SNP14 at LOD score of 6.78, 8.31, 34.39, 9.1, 2.31; +3027 SNP5, (rs17179101) with SNP-7, SNP-12, SNP-13 (LOD, 18.46, 3.61, 2.44); +3035 SNP-7 (rs17179108) with SNP12, SNP13 at LOD score of 10 and 2.03.The SNPs - 8,-9,-10 were monomorphic. The +3142 SNP12 showed LD with SNP13 (LOD, 9.46).

In the HCC group (**Table 5**B), LD was observed for +2960 SNP1 (rs371194629) with SNP4(rs1710), SNP5 (rs371194629), SNP7 (rs17179108), SNP12(rs1063320) and SNP14 (rs1610696) at LOD, 4.46, 8.41, 9.58, 4.98 and 2.37. SNP-2, -6, -8, and -11 had shown no linkage with any other SNPs. The +3003 SNP3 (rs1707) had shown linkage with SNP-4, -5, -7, -12, and-13 at LOD scores of 11.26, 6.16, 6.88,17.31 and 4.51; and +3010 SNP4 (rs1710) with SNPs -5, -7, -12, -13, and -14 at LOD scores of 10.97, 12.85, 28.09, 5.21, and 2.53. + 3027 SNP5 (rs17179101) showed linkage with SNPs -7, -12, and -13 with LOD scores of 22.75, 12,99, 2.39; and +3035 SNP7 (rs17179108) with SNP-12 and SNP-13 (LOD, 12.64, 2.6). The +3092 SNP9 (rs180827037) and SNP10(rs760052251) were monomorphic. +3142 SNP-12 (rs1063320) showed LD with SNP-13 and -14 (LOD, 4.45 and 2.98).

**Table 5:** Logarithm of odds (LOD) score and correlation (r^2^) value among 14 SNPs showing non-random association with each other among healthy and HCC population.

A. Healthy population, LOD score and r2 value														
SNP	1	2	3	4	5	6	7	8	9	10	11	12	13	14
**1. rs371194629**	1	1.97,0.145	0.86.0.02	**3.81, 0.09**	**4.67,0.09**	0.02.0.01	**9.68,0.18**				0.63,0.07	**3.28, 0.079**	0.93,0.023	0.47,0.01
2. rs567747015	0.16, 0.02	1	0.95.0.028	0.04,0.001	1.84,0.02	0.11,0.001	1.04,0.016				**6.2,0.329**	0.79.0.02	0.09,0.002	0.1.0.002
**3. rs1707**	1.73, 0.05	0.59,0.021	1	**7.61,0.18**	0.96,0.017	0.0, 0.0	3.87,0.062				1.01,0.04	**17.18,0.346**	1.32,0.036	0.29,0.007
**4. rs1710**	4.46, 0.113	0.01,0.0	**11.26,0.279**	1	**6.78,0.096**	0.41,0.006	**8.31,0.119**				0.01.0.0	**34.39,0.65**	**9.1,0.22**	**2.31,0.035**
**5. rs17179101**	**8.41, 0.173**	0.67,0.012	**6.16,0.09**	**10.97,0.203**	1	0.33,0.01	**18.46,0.517**				0.92,0.011	**3.61,0.067**	**2.44,0.033**	0.56.0.007
6. rs146339774	0.01, 0.001	0.18,0.01	0.01,0.0	1.1,0.02	0.01,0.001	1	0.06,0.003				0.36,0.025	0.08,0.011	0.2,0.006	0.75.0.043
**7. rs17179108**	**9.58, 0.206**	0.21,0.005	**6.88,0.112**	12.85**,0.238**	**22.75,0.708**	0.62,0.03	1				0.83,0.013	**10.0,0.166**	**2.03,0.034**	0.02,0.001
8. rs569057854	0.06,0.006	0.59,0.034	**0.12,0.008**	0.36,0.007	0.06,0.001	0.01,0.0	0.07,0.001	1						
9. rs180827037									1					
10.rs760052251										1				
11.rs138249160	0.62, 0.06	**1.62,0.101**	1.5,0.046	**0.03,0.001**	0.28,0.007	0.31,0.024	0.37.0.009	0.02,0.0			1	**1.65,0.052**	0.06.0.002	1.15, 0.043
**12.rs1063320**	**4.98, 0.129**	0.43, 0.013	**17.31, 0.36**	**28.09,0.621**	**12.99,0.266**	0.07,0.002	**12.64,0.28**	0.2,0.003			1.26.0.028	1	**9.46,0.204**	0.26,0.006
13.rs9380142	2.08, 0.052	0.0, 0.0	4.51,0.12	**5.21,0.111**	**2.39,0.034**	0.09,0.001	**2.6,0.037**	0.09,0.001			0.52,0.011	4.45,0.086	1	0.0,0.0
**14.rs1610696**	**2.37,0.051**	0.0, 0.0	1.26,0.025	2.53,0.056	0.44,0.006	0.02,0.0	0.48,0.006	0.02,0.0			0.15,0.002	**2.98,0.07**	0.63,0.009	1
B. HCC population, LOD score and r2 value														

High LOD score (LOD ≥ 2) indicates strong linkage, whereas a high r^2^-value (r^2^ > 0.6) indicates strong correlation between two SNPs. SNPs are indicated as numbers 1-14, Top panel (A) is for healthy population and bottom panel for HCC patient. Each column represents both LOD score and r^2^-value and strong correlation between SNPs are indicated in bold.

The bold values indicate strong linkage (LOD>2).

### HLA-G UTR haplotype determination

We used haploview to look at the haplotypes of all 14 SNPs (**Figures 3A–C**). For the Indian population, there were 14, 18, and 16 different haplotypes in healthy, HCC, and among all total recruited participants, respectively, with frequencies above 1%. Castelli et al. (24) proposed UTR typing using 8 SNPs that include SNP1 (+2960), SNP3 (+3003), SNP4 (+3010), SNP5 (+3027), SNP7 (+3035), SNP12 (+3142), SNP13 (+3187), and SNP14 (+3196) in the UTR region (24). Using Haploview, both the LD (**Figures 3D–F**) and haplotype analysis were carried out for these 8 SNPs (**Tables 6**A–C). In the healthy, HCC, and mixed populations, these eight SNP haplotype analyses found 12, 13, and 13 distinct haplotypes that present with a frequency > 1 percent among the population (**Table 6**A). Till now 45 different UTR types were named as per 8 SNPs haplotypes;UTR1-8 (24), UTR 9-16 (25) or as per all 16 SNPs haplotypes that include UTR 17-45 (26). Prevalent UTR types in the healthy population were UTR3 (0.195), UTR15 (0.137), UTR4 (0.126), UTR1 (0.113), and UTR7 (0.109). Whereas in the HCC population (**Table 6**B), prevalent UTR types were UTR 4 (0.239), UTR1 (0.126), UTR7 (0.12), and UTR3 (0.112). The haplotype using 14 SNPs can produce different UTR haplotypes (UTR1-45). The UTR haplotypes including UTR1-18 named using the initial 8 SNPs, may overlap with other UTR haplotypes those determined using 14 SNPs such as UTR-1/29/36, UTR-3/23/37/39, UTR-4/27, UTR-5/17/33/44, and UTR-6/18/20/35. Therefore, we named five new observed haplotypes with >1% frequencies as UTR-46 (ICGCCCAC), UTR-47 (ICCCCCAC), UTR-48 (DTCCCCAC), UTR-49 (ITGCCCAC) and UTR-50 (ITCCCCAC). We have also observed eight different haplotypes containing insertions of 14 bp.

**Table 6:** Prevalent haplotype and UTR types with frequency > 1% in the healthy (A), HCC (B) and mixed total (C) population.

A. Healthy	B. HCC	C. Total
Haplotype Freq N UTR type* DTCCCGAC 0.195 43 UTR3 ITCCCGAC 0.137 30 UTR15 DCGCCCAC 0.126 28 UTR4 DTGCCCGC 0.113 25 UTR1 ITCATGAC 0.109 24 UTR7 ICGCCCAC 0.048 11 UTR46(New) ITGCCCGC 0.043 9 UTR30 ITCCTGAC 0.041 9 UTR5 ICCCCCAC 0.029 6 UTR47(New) DTGCCCAC 0.029 6 UTR6 ITCCCGAG 0.027 6 UTR2 ITCCCCAC 0.013 3 UTR50(New)	Haplotype Freq N UTR type* DCGCCCAC 0.239 48 UTR4 DTGCCCGC 0.126 25 UTR1 ITCATGAC 0.120 24 UTR7 DTCCCGAC 0.112 22 UTR3 ICGCCCAC 0.097 19 UTR46(New) ITCCCGAC 0.040 8 UTR15 ITCCCGAG 0.037 7 UTR2 ICCCCCAC 0.034 7 UTR47(New) DTCCCCAC 0.029 6 UTR48(New) ITGCCCAC 0.029 6 UTR49(New) ITGCCCGC 0.028 6 UTR30 DTGCCCAC 0.026 5 UTR6 ITCCTGAC 0.020 4 UTR5	Haplotype Freq N UTR type* DCGCCCAC 0.181 76 UTR4 DTCCCGAC 0.149 63 UTR3 DTGCCCGC 0.116 49 UTR1 ITCATGAC 0.115 48 UTR7 ITCCCGAC 0.097 41 UTR15 ICGCCCAC 0.071 30 UTR46(New) ITGCCCGC 0.040 17 UTR30 ITCCCGAG 0.034 14 UTR2 ICCCCCAC 0.032 13 UTR47(New) ITCCTGAC 0.031 13 UTR5 DTGCCCAC 0.027 11 UTR6 DTCCCCAC 0.023 10 UTR48(New) ITGCCCAC 0.019 8 UTR49(New)

*UTR1: Overlaps with UTR-29, -36; UTR2: overlaps with UTR-26, UTR-32 for 8 SNPs

UTR3: overlaps with UTR- 23, -37, -39; UTR 4: overlaps with UTR-27;

UTR5: overlaps with UTR-17, -33 and -44; UTR6: overlaps with UTR-18, -20, -UTR-35.

### Comparison of sHLA-G levels as per tumor size, BCLC stage, SNP genotype, and UTR haplotype in HCC patients and healthy population

**Figure 4:**
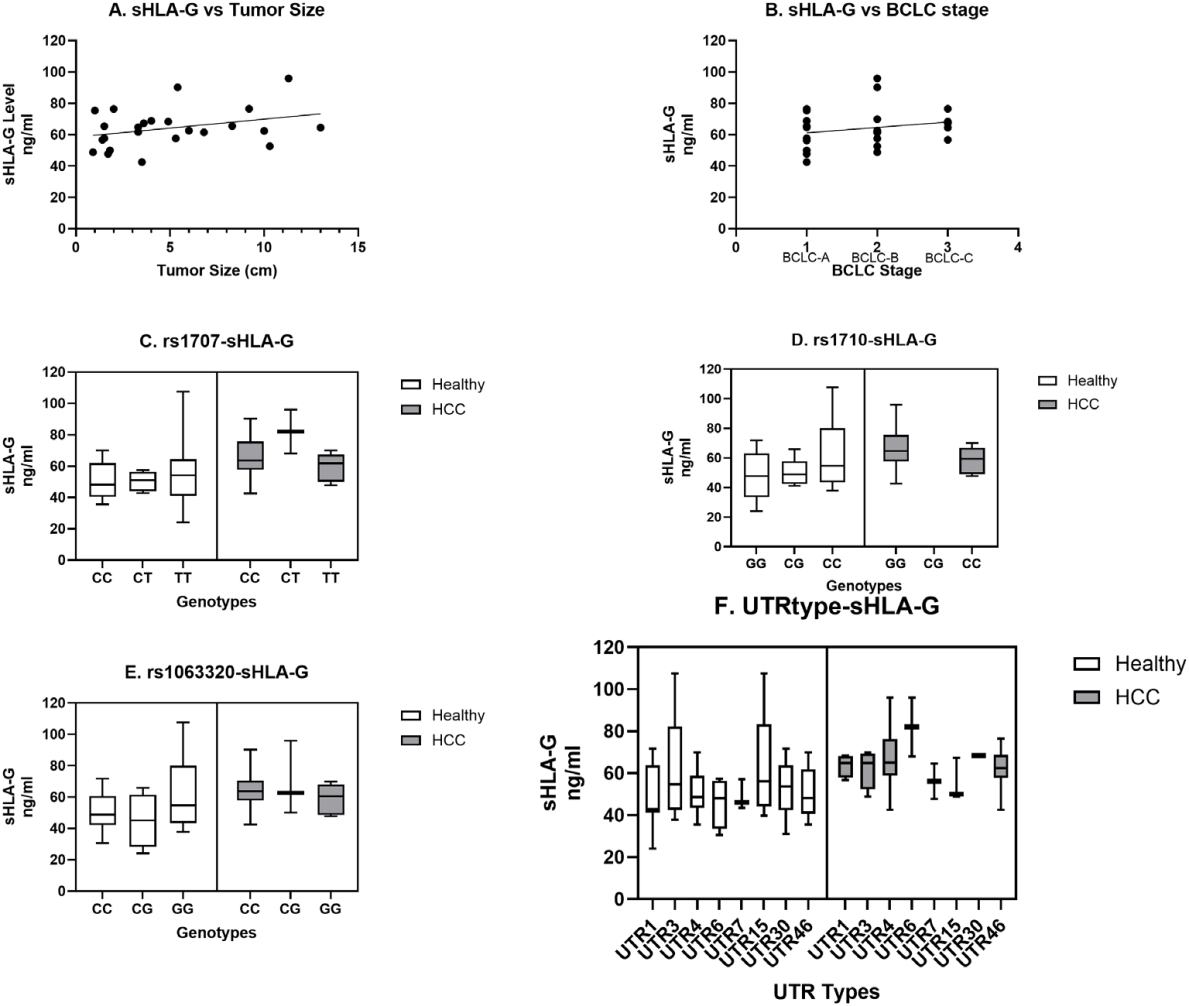
Correlation of sHLA-G level with tumor size, stage, prevalent SNP genotypes and UTR types. Increasing trend in sHLA-G level was observed with tumor size **(A)** and BCLC stage **(B)**. Comparison of SNP genotypes **(C–E)** and UTR types **(F)** for sHLA-G level was carried out for SNPs rs1707 **(C)**, rs1710 **(D)**, rs 1063320 **(E)** and UTR types **(F)** in HCC and healthy population.

We have observed an increased level of sHLA-G in HCC patients as compared to the healthy population (**Figure 1**). When sHLA-G levels were compared in patients with different tumor sizes, an increased trend and positive correlation were observed with tumor size (r = 0.32; **Figure 4A**). Similarly, a stage-specific increased trend with sHLA-G level was observed with a positive correlation value (r = 0.22) (**Figure 4B**). This clearly indicates that sHLA-G level may increase as per advanced BCLC stage or bigger size tumor in HCC patients. Out of 14 SNPs, we have observed significant genetic association with HCC for SNPs rs1707 (C>T), rs1710 (G>C), and rs1063320 (C>G) (**Table 3**). We have observed increased sHLA-G levels in HCC patients as compared to healthy controls, irrespective of different SNP genotypes (**Figures 4C–E**). However, we observed an increased trend in sHLA levels in HCC patients with the risk CC genotype/C allele for SNP rs1707 (**Figure 4C**), the risk GG genotype or G-allele for SNP rs1710 (**Figure 4D**), and the risk CC genotype or C-allele for SNP rs1063320 (**Figure 4E**). An increased level of sHLA-G was observed in HCC patients for diplotypes or haplotypes containing UTR4>UTR3>UTR1>UTR46, whereas in healthy individuals, increased levels were observed for UTR3>UTR15>UTR30>UTR4>UTR46 (**Figure 4F**).

## Discussion

Human leukocyte antigen-G (HLA-G) is a new nonclassical MHC class I protein that is different from classical HLA class I molecules because it has low polymorphism, less protein variability, and a limited tissue distribution. HLA-G is known to suppress the cytotoxic action of CD8 and NK cells, inhibit antigen presentation, and promote lymphocyte proliferation via communication with ILT-2 and ILT-4 leukocyte receptors (27, 28). HLA-G, along with IL-10 expressing dendritic cells, can induce regulatory T cells (29). HLA-G is commonly expressed in the fetus and protects it from the maternal immune response (30). But in healthy non-fetal subjects, its expression is limited to the cornea, thymic medulla, nail matrix, beta cells of the islets of Langerhans, mesenchymal stem cells, and endothelial precursors (31). Even though HLA-G expression is restricted to a few tissues in normal conditions, it increases robustly in pathological conditions. HLA-G function has also been reported to maintain tolerance in autoimmune and inflammatory diseases and post-transplantation, as well as mediate immune escape in cancer and infectious diseases (32). Therefore, HLA-G is considered a tolerogenic and immune checkpoint molecule, which plays a dual role of immune evasion for tumors and allograft’s acceptance for solid organ transplant (33). Studies have reported the involvement of HLA-G in various cancers, including HCC. Cai and coworkers had shown that HLA-G expression was found to be associated with the prognosis of HCC, reduced overall survival, and increased tumor recurrence (34). In our study, levels of serum HLA-G were found to be elevated in HCC cases as compared to healthy control. This was supported by a study where HCC patient’s HLA-G levels in serum and biopsy specimens were greater than controls, confirming the potential diagnostic role of HLA-G. In addition, 50% of HCC cases out of a larger cohort were HLA-G positive (34). Another study also reported a positive correlation of HLA-G with patients age and disease stage (35). One similar study indicated that higher serum expression of HLA-G in HCC patients as compared to healthy controls (36). We have also observed increased serum levels of HLA-G in HCC patients, which can significantly differentiate healthy from HCC using the ROC curve (**Figures 1A, B**). An increased level of HLA-G soluble form (35 kDa) was found in the serum of HCC patients as compared to healthy control (**Figures 1C, D**). Park et al., 2012 have shown increased sHLA-G expression in active HBV and HCC groups indicating its diagnostic relevance during infection and HCC progression (37). In another study, the areas under the receiver-operating characteristic (ROC) curves recommended sHLA-G as a marker for differential diagnosis of various malignancies against healthy controls (38).

The HLA-G gene is located within the MHC region at chromosome 6p21.3, which consists of 8 exons and 7 introns (39, 40). Due to presence of a stop codon in exon 6, exon 7 is lost from mature mRNA, and exon 8 remains untranslated (41). The HLA-G gene expresses seven isoforms, including four membrane-bound (G1 to G4) and three serum-soluble (G5 to G7) proteins. The soluble isoform HLA-G5 is secreted actively, and the membrane-bound HLA-G1 is also released into the secretion by proteolytic cleavage by matrix metalloproteinase-2 (42). The HLA-G gene per se is less polymorphic at the coding region as compared to other HLA genes. The genetic diversity of HLA-G coding region is indicated as HLA-G alleles those are reported through IPD-IMGT/HLA database (43). As of December 2023, at the website https://hla.alleles.org/nomenclature/stats.html, 158 HLA-G alleles were reported that encode 48 different proteins, and among them are six null alleles (43). Other studies reported 190 different alleles, of which only 14 HLA-G alleles were reported to show global frequencies >1%; those include G*01:01:01:01, G*01:01:01:04, G*01:01:01:05, G*01:01:01:06, G*01:01:01:08, G*01:01:02:01, G*01:01:03:03, G*01:01:22:01, G*01:03:01:02, G*01:04:01:01, G*01:04:01:02, G*01:04:04, G*01:05N, and G*01:06:01:01 (44). The polymorphisms at both 5’ upstream regulatory promoter region (1.4 kb from the ATG initiation codon, 5′ URR) and the 3’ untranslated region (UTR) modulate HLA-G expression (9). The genetic polymorphisms at 3’ UTR SNPs mostly affect microRNA binding, alternate splicing and mRNA stability thereby influence HLA-G expression. In our study, we first looked at how the genotype, allele frequency (**Table 2**), and haplotype structure (**Figures 3**, **4**) of all 14 UTR SNPs might be different between groups of healthy controls and HCC patients of Indian ethnicity. Then, the genetic associations of HLA-G UTR polymorphisms were analyzed from the perspective of HCC risk and its treatment response to locoregional therapy (**Table 4**). The most common UTR haplotypes prevalent among groups were determined. HWE testing was carried out for all 14 causal SNPs, and the test of deviation for 4 out of 14 SNPs (+2960 SNP1, +3001 SNP2, +3032 SNP6, +3125 SNP11) was found to be non-significant (**Table 2**), which may be considered good genetic traits as they are not under the influence of any evolutionary pressure (45). Three SNPs (+3052 SNP8, +3092 SNP9 and +3120 SNP10) were monomorphic and not analyzed for genetic association. Assuming that the individual UTR SNP allele and genotype are disease traits, a genetic association with a significant OR>1 is thought to be a risk for HCC or a non-response, while an OR<1 is thought to be a protective (**Table 3**). The genetic association with OR>1 was observed for three SNPs which include + 3003 SNP3 (rs1707, C-allele, OR:1.93, 95% CI-1.25-2.95), +3010 SNP4 (rs1710, G-allele, OR: 2.14, 95% CI-1.44-3.14), and +3142 SNP12 (rs1063320, C-allele, OR: 2.00, 95% CI-1.35-2.94). These three SNPs are important for miRNA binding in the UTR region. For these three SNPs, minor allele was found to be risk allele (OR>1) and the major allele can be considered as protective. The miRNA usually represses translation; therefore, dysregulation miRNA targeting these SNP alleles may modulate HLA-G expression, increasing the risk for HCC. In cancer, miRNA dysregulation also occurs and we had also reported dysregulation of miRNA in HCC (46). The miRNAs (miR-654-5p, miR-541-3p, miR-3158-5p, miR-4492, and miR-4498; miR-4800-5p, miR-7705, miR-3619-3p, miR-6854-5p, and miR-767-5p) predicted to bind HLA-G UTR containing rs1707, rs1710, and rs1063320 SNP alleles were found to be dysregulated in HCC as per published literature (47–49). We did not observe significant association of most studied +2960 14-bp Indel polymorphisms with HCC. The presence of 14bp insertion (5´-ATTTGTTCATGCCT-3´) in 3´UTR is known to influence the mRNA stability and generate an additional splice whereby 92 bases are removed from the start of exon 8 (50). These 92 bases deleted alternative transcript, is more stable, is associated with increased HLA-G soluble levels. The genetic association with this 14 bp Indel was observed in many different types of cancer. There are also report suggesting reduced generation of most HLA-G membrane and soluble isoforms in persons carrying 14 bp insertion allele (50). Whereas studies have also shown deletion allele to be more frequent in HCC and might contribute to higher HCC receptiveness (51). We have also higher frequency of 14 bp deletion allele in HCC patients as compare to healthy control (**Table 2**). Similarly in case of breast cancer, digestive tract tumors and HCC 14-bp, Del allele was associated with disease susceptibility as reported by Dias et al. (52). Coelho et al., 2016 also demonstrated no significant association of 14-bp ins/del SNP with HCC In a meta-analysis and suggested possible role of other UTR SNPs (53).

We have derived haplotypes from all 14 SNPs and from 8 SNPs and UTR types in healthy control, HCC cases and combined populations were named as per earlier published literature (24–26). The LD pattern was plotted for all 14 or defined 8 SNPs to assess whether alleles at all these SNP loci tend to be inherited collectively or if there is a chance of any ancestral recombination events, and what is the population divergence among healthy controls, HCC patients, and combined populations (45). We have observed three monomorphic SNPs in the UTR region. Like our result, a monomorphic LD pattern was observed for +3032 SNP8 in Asian and American populations and for +3092 SNP9 in Europe, Asia, and the American population of 1000 genome data (26). The most frequent global UTR haplotypes are UTR-1 and UTR-2. Initial haplotypes (UTR1-16) were reported in Brazilian population based on 8 SNP loci in UTR region (24, 25). Based on all 16 SNP sites, a total of 45 (UTR1-45) haplotypes were inferred using 1000 Genomes data of 21 worldwide populations (26). When compared with these 45 haplotypes, we found 5 new haplotypes with frequencies more than 1% named as UTR-46 to -50. We observed a varied pattern of the top five most frequent haplotypes that are different in the healthy (UTR-3> UTR-15> UTR-4> UTR-1> UTR-7), HCC (UTR-4> UTR-1> UTR-7> UTR-3> UTR-46), and combined (UTR-4> UTR-3> UTR-1> UTR-7> UTR-15) populations in India (**Figure 4**). Recently, Drabbels et al. (54) showed high linkage disequilibrium between UTR haplotypes and the coding sequence, or HLA-G alleles (54). The UTR-3 haplotype, which is highly prevalent in Indian healthy controls, was found to be strongly associated with the HLA-G*01:04 allele and low levels of soluble HLA-G in a separate study (54, 55). The UTR-4 and UTR-1 haplotypes which is highly prevalent in Indian HCC patients found to be strongly associated with G*01:01:01 allele and considered as high producers of soluble HLA‐G in another study (54, 56). The UTR-7 haplotype was also found to be linked with the G*01:01:03 allele and is considered a low producer of sHLA-G (54, 56). In another study, the UTR-4 haplotype among 7 haplotypes was found to be a risk factor for prostate cancer when compared to the control (57).

Several studies related to HLA-G UTR polymorphism in India were carried out for different ethnic regions, including north India, south India, and north-east India. Most of these studies were limited to the +2960 Indel and +3142 G/C polymorphisms. One study related to head and neck squamous cell carcinoma (HNSCC) from Northeast India was conducted by exon 8 sequencing data observed UTR-5 and UTR-7 as most frequent haplotypes (58). We have also observed UTR-5 and -7 haplotype in our study. In another study from south Indian population related to diabetes had shown increased +2960 14bp Del allele and 3142 G allele (59). Higher frequency of +2960 Del allele and +3142 C allele was observed in bipolar disorder cases than controls from South India (60). Other study from South India had shown significant increased frequency of +2960 14bp Ins allele and +3142 G allele in breast cancer patients than healthy controls (61). Other study from north India had shown significant association and increased frequencies of +2960 14bp Ins allele and +3142 C allele in HNSCC patients (62). In our study from north Indian population significant association was not found for 2960 Indel polymorphism but +3142 C-allele found association with HCC.

In conclusion, our study found a genetic association between HLA-G UTR polymorphisms and HCC and treatment responses. We had also observed higher sHLA-G levels in HCC patients than healthy controls. We have observed an increased frequency of the UTR3 haplotype in healthy controls and an increased frequency of the UTR-4 and UTR-1 haplotypes in HCC patients. We have not observed effects of 5’ URR polymorphism and coding sequence or HLA-G allele in HCC, which is a limitation of our study. This study is the first to show the genetic association of HLA-G with HCC in the Indian population.

## Data Availability

The datasets presented in this study can be found in online repositories. The names of the repository/repositories and accession number(s) can be found below: GenBank with accession number spanning from PP278638 to PP278737 for HCC patients and the accession number spanning from PP278738 to PP278847 for healthy controls.
